# pH stimuli-responsive hydrogels from non-cellulosic biopolymers for drug delivery

**DOI:** 10.3389/fbioe.2023.1270364

**Published:** 2023-09-12

**Authors:** Udaykumar Vegad, Megha Patel, Dignesh Khunt, Ožbej Zupančič, Sanjay Chauhan, Amrit Paudel

**Affiliations:** ^1^ Graduate School of Pharmacy, Gujarat Technological University, Ahmedabad, Gujarat, India; ^2^ Research Center Pharmaceutical Engineering GmbH (RCPE), Graz, Austria; ^3^ Institute of Process and Particle Engineering, Graz University of Technology, Graz, Austria

**Keywords:** non-cellulosic biological macromolecules, pH-sensitive polymer, hydrogel, drug delivery, formulation

## Abstract

Over the past several decades, there has been significant growth in the design and development of more efficient and advanced biomaterials based on non-cellulosic biological macromolecules. In this context, hydrogels based on stimuli-responsive non-cellulosic biological macromolecules have garnered significant attention because of their intrinsic physicochemical properties, biological characteristics, and sustainability. Due to their capacity to adapt to physiological pHs with rapid and reversible changes, several researchers have investigated pH-responsive-based non-cellulosic polymers from various materials. pH-responsive hydrogels release therapeutic substances in response to pH changes, providing tailored administration, fewer side effects, and improved treatment efficacy while reducing tissue damage. Because of these qualities, they have been shown to be useful in a wide variety of applications, including the administration of chemotherapeutic drugs, biological material, and natural components. The pH-sensitive biopolymers that are utilized most frequently include chitosan, alginate, hyaluronic acid, guar gum, and dextran. In this review article, the emphasis is placed on pH stimuli-responsive materials that are based on biological macromolecules for the purposes of drug administration.

## Graphical Abstract

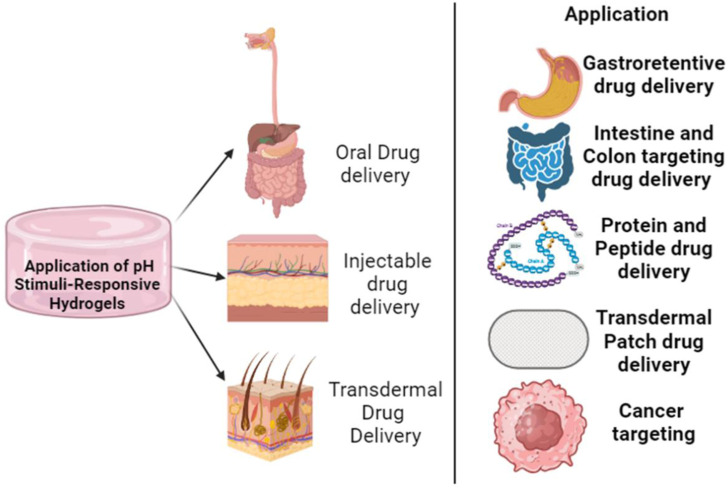

## 1 Introduction

In recent years, there has been an increase in interest in personalized pharmacotherapy and precision medicine, both of which have been the driver for smart biomaterial design. Stimuli-responsive hydrogels can be considered as smart biomaterials, and external triggers like as pH, temperature, electrical and magnetic fields, light, and biomolecule concentration can be employed to elicit drug release (Salehi et al., 2023). Hydrogels are three-dimensional polymeric systems with considerable promise for application as transport conduits for medicines, bioactive components, and dietary ingredients. Hydrogel has the potential to accommodate enormous volumes of water through capillary action and osmotic pressure. These characteristics make hydrogels resemble extracellular matrices in living tissues (Aswathy et al., 2020). Hydrogels made from sustainable biological macromolecules have a number of beneficial properties, including low immunogenicity, excellent biocompatibility and biodegradability, cytocompatibility, cellular/tissue targeting, stability, superb structural design, 3D geometry, and tunable solubility (Gu et al., 2022). Some limitations of bio-macromolecular hydrogels include their natural origin and thus the batch-to-batch variability, fragile mechanical strengths, limited processability (for example, chitosan), source limitations, rapid biodegradation, rapid catabolization rates (for example, gelation), as well as microbial degeneration (especially in the case of polypeptides) (Abbasian et al., 2019; Salehi et al., 2023).

Recently, pH-sensitive hydrogels have been extensively investigated and employed in biomedical applications, particularly in drug delivery systems that take advantage of pH shifts along the gastrointestinal tract (GIT) and targeted delivery of specific therapeutics such as anticancer drugs and genes (Rizwan et al., 2017; Salehi et al., 2023). pH-responsive hydrogels possess a notable advantage in the field of drug delivery due to the presence of pH fluctuations in various physiological compartments along the physiological pH ranges such as the gastrointestinal tract, tumor microenvironment, and intracellular compartments. pH-responsive hydrogels possess the capability to be deliberately designed and tailored to exhibit a site-specific response to particular pH ranges. This unique characteristic enables controlled drug release in these specific pH environments (Salehi et al., 2023). pH-responsive hydrogels provide controlled drug release kinetics via pH-dependent swelling or collapse behavior. Such customization of drug release profiles can be achieved by modifying the hydrogel composition and crosslinking density, thereby accommodating specific therapeutic needs (Rizwan et al., 2017; Bami et al., 2022; Ding et al., 2022).

As cellulose-based pH-sensitive hydrogels have been extensively reviewed previously and have been thus, (Chen et al., 2019; Fu et al., 2019; Deng et al., 2022), excluded from the scope of this review. Biopolymer-based materials that are sensitive to various stimuli, including pH, temperature, light, electrical or magnetic fields, and ionic strength have been developed. However, pH and temperature are two principal stimuli that exist naturally in the human physiological environment (Zhuo et al., 2020; Cui et al., 2021).

The use of pH-responsive hydrogels derived from non-cellulosic biological macromolecules is an emerging area of research that lies at the intersection of materials science and medication delivery. Due to their intrinsic pH sensitivity, these hydrogels have significant potential in the domains of controlled drug release and targeted therapy. The development of hydrogel systems with adjustable pH-triggered behaviors has resulted in the ability to precisely control drug delivery profiles in accordance with certain physiological conditions. Hydrogels have been used in many therapeutic domains, including cancer therapy and oral drug administration, with the aim of augmenting treatment effectiveness while mitigating adverse reactions (Garshasbi et al., 2023). Nevertheless, there are still obstacles that need to be overcome in order to achieve the desired mechanical qualities, ensure long-term stability, and address possible issues with the immunogenicity of biological macromolecules. The potential of integrating pH-sensitive hydrogels with other responsive stimuli, such as temperature or enzymes, to develop multifunctional delivery platforms, presents promising avenues for future research. Furthermore, the tailoring of hydrogels to align with specific patient profiles has the potential to advance personalized medicine, hence driving the field towards therapies that prioritize the needs and characteristics of particular patients. In the current review, intrinsic physio-chemical properties and application of non-cellulosic sustainable pH stimuli-responsive macromolecule hydrogels are discussed.

## 2 Properties of non-cellulosic biological pH-sensitive polymer

pH-sensitive polymers alter their structure and characteristics, such as surface activity, chain conformation, and solubility, in response to pH changes. pH and functional groups regulate the characteristics of pH-sensitive hydrogels (Bami et al., 2022). pH-sensitive block copolymers or network structured pH-sensitive polymers react to pH changes by self-assembling into unimers, micelles, gels, vesicles, swelling/deswelling phenomena, etc. as a function of their degree of (de)protonation (Kocak et al., 2016). Chitosan, alginate, hyaluronic acid, dextran, xanthan, and gum-tragacanth are some examples of pH-sensitive biopolymers (Belscak-Cvitanovic et al., 2015; Garshasbi et al., 2023).

**FIGURE 1:**
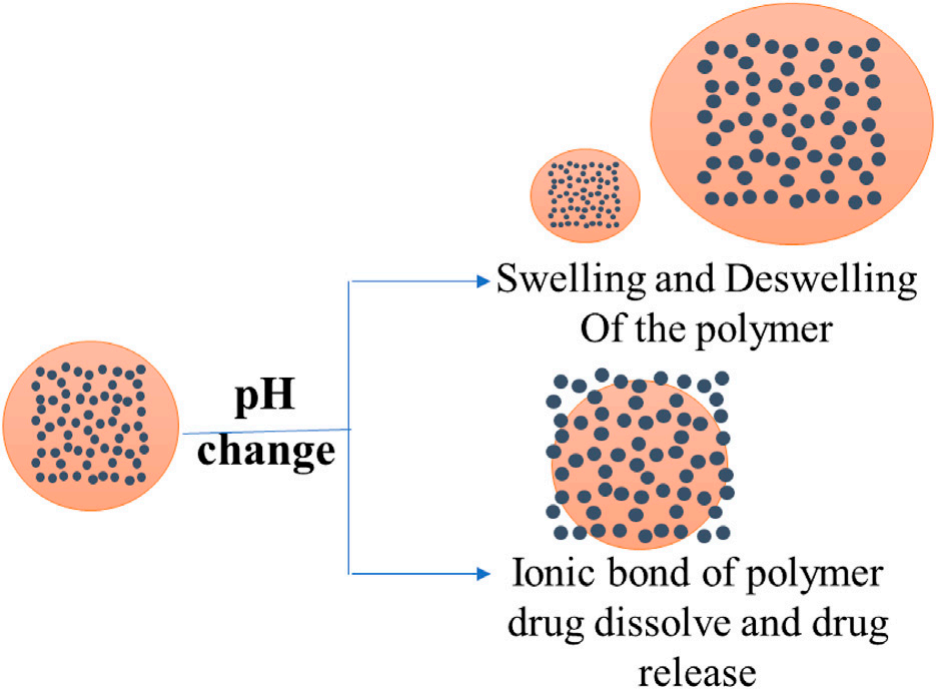
Schematic representation of two basic approaches used in the formation of pH-responsive drug delivery.

**FIGURE 2:**
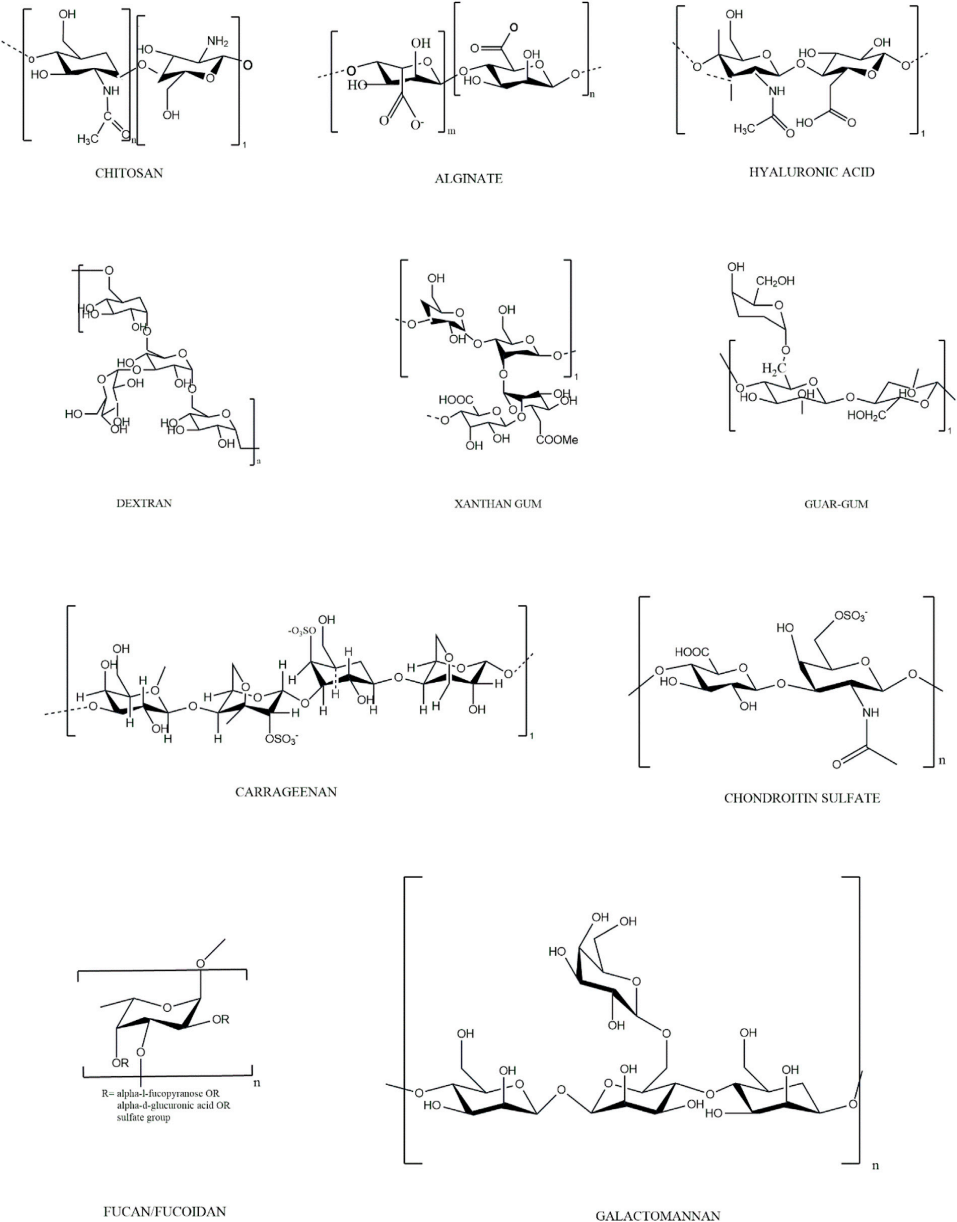
Structures of monomeric units of Non-cellulosic Biological Macromolecules.

Ionic hydrogels with charge-carrying functional groups are responsible for pH-sensitive swelling. They are influenced by various factors, including ionic charge (strength and size), dissociation constants (pK_a_ or pK_b_), ionization degree, hydrophilicity, polymer concentration, and swelling media pH. A polymer with acidic functional groups reveals an expanded state when the medium pH is greater than pKa, while it dismantles at pH < pK_a_. Likewise, polymer chain dismantling for a polybase occurs above the value of pK_b_ (pH > pK_b_) (thus, they expand at pH < pK_b_). Cationic polymers like chitosan swell in pH media below pK_b_ due to the protonation of cationic amine/imine group (Ding et al., 2022; Rehman et al., 2022). The hydrogenated positively charged groups lead to swelling of the polymer chains, creating repulsion between functional groups. As such, they can be employed as carriers for parenteral drug delivery systems or targeted gastric delivery of antibiotics in the treatment of ulceritis. Anionic polymers like sodium alginate swell at basic pH values because of the ionization of the acidic groups. Thus, the negative charge of polymer chains induces a repulsion between themselves, resulting in facilitated swelling. This feature of hydrogels can be utilized for colon drug delivery at pH of 7.4 (Du et al., 2015). Non-cellulosic biological macromolecule based pH-sensitive polymers can be distinguished by their origin. A pH-responsive structural conformation with solubility variations in biopolymers is a common issue (Gil and Hudson, 2004). The drug delivery mechanism of pH-responsive hydrogel is demonstrated schematically in Figure 1. The monomeric units of pH-sensitive non-cellulosic polymers are listed in Table 1 and Figure 2.

**TABLE 1:** Properties of the Non-cellulosic Biological Macromolecules with their advantages.

Name of Polymers>	Monomer units	Ionic nature	Source	pK_a_	Advantages
Chitosan	D-glucosamine and N acetyl-D-glucosamine	Cationic	Exo-skeleton of crustaceans (shrimps, crabs, lobsters), yeast and fungi	6.5	low toxicity, bioactivity, biodegradability, antibacterial properties, hemostatic effect, and excellent mucoadhesive properties
					potential adjuvant to vaccine and modifier to macrophage clearance
Alginate	D-mannuronic acid (M) and -L-guluronic acid (G)	Anionic	Brown seaweed	3.2	high content of acidic functional groups
					forms a gel having different swelling characteristics in the presence of divalent cations such as Ca2+, Ba2+, Sr2+, and Zn2+
					permit the encapsulation of different compounds or even cells
Hyaluronic acid	D-glucuronic acid and N-acetyl-glucosamine disaccharide units linked by β (1, 4) and β (1, 3) glucosidic bonds	Anionic	Part of almost every tissue in vertebrates	3–4	swells up to 1000-fold
					anionic polyelectrolyte at neutral pH, rendering it very hydrophilic
Dextran	D-glucose linked by 1,6-glycosidic bonds	Cationic	Bacteria (Leuconostocmesenteroides)	7.4	dextranase generated by colon bacteria can break it
Xanthan	D-glucose (linked through 1,4-glycosidic linkage) and a trisaccharide branch connected by 1,3-glycosidic bonds on the backbone’s alternating glucose units with 2:2:1 ratio of D-glucose, D-mannose, and D-glucuronic acid	Anionic	Bacteria (*Xanthomonas compestris*)	3.1	produced by anerobic bacteria *Xanthomonas compestris*
Guar-gum	D-mannopyranose (-D-mannose) units linked to the main backbone through 1, 4-glycosidic bonds, with periphery branches of -D-galactopyranose (-D-galactose) at every alternate mannose unit attached to the main backbone with 1, 6-glycosidic bonds	Non-ionic	Endosperm of Cyamopsistetragonolobus	3.5	biodegradable, non-toxic, readily commercially available, hydrophilic nature
Carrageenan	D-galactose and -D-galactose or 3,6-anhydro—D-galactose linked by −1,3 and −1,4-glycosidic links	Anionic	Cell wall of Red seaweed	4.9	quantity of sulphate substitution and the equilibrium of related cations determine carrageenan water solubility, viscosity, and gel strength
Chondroitin sulfate	(1–3)-β-N-acetyl- D-galactosamine and (1–4)- β -glucuronic acid	Anionic	cartilage, bone and cornea of animals	−3.7	strongly hydrophilic and lack of mechanical stability - needs to be chemically functionalized or used with other polymers for drug delivery and tissue engineering applications
Fucan and fucoidans	backbones consisting of (1,3)-linked-l-fucopyranose residues or backbones with alternating (1,3)-linked and (1,4)-linked l-fucopyranose residues, with sulfation at varying degree	Anionic	Brown Seaweed—*Fucus* spp.	1.0–2.5	Water soluble sulphated polysaccharide—the degree of sulfate substitution determines the characteristic properties of the polymer
Mannan and Galacotmannan	Mannose linked by β (1–4) linkage and galactose in side chain linked by α (1–6) linkagae	Neutral	Endosperm of leguminous seeds		high molecular weight, high water solubility, and a hydroxyl-rich side chain—helps in intramolecular crosslinking and overall network entanglement, - imparts mechanical properties

Chitosan, the second most prevalent polymer after cellulose, is made of D-glucosamine and N acetyl-D-glucosamine residues from chitin deacetylation. (Drury and Mooney, 2003). Chitosan is widely applied due to its low toxicity, bioactivity, biodegradability, antibacterial, hemostatic, and mucoadhesive qualities (Costa-Pinto et al., 2011; Verma et al., 2017). Chitosan’s antimicrobial activity is related to two mechanisms: (i) cationic polymers of chitosan interact with sialic acid in phospholipids in the microbial cell membranes, limiting their mobility and disrupting the net negatively charged cell membrane structure and (ii) oligomeric chitosan enters microorganism cells and converts DNA to RNA, stopping growth (Sashiwa et al., 2004). Apart from that, Chitosan shows strong mucoadhesive properties due to its cationic nature and interaction with net negatively charged intestinal mucus layer, enhancing its residence time in the colon and drug diffusion gradient, leading to increased absorption (Shah et al., 2016). The mechanism of mucoadhesion lies in interactions between positive chitosan with negatively charged sialic acid and sulphate groups present in mucin residues in the mucus layer, (Du et al., 2015). In addition, chitosan is a potential vaccination adjuvant and a macrophage clearance modifier due to its mucoadhesive properties, especially in the nanoparticle form (Wei et al., 2017).

Alginate from brown seaweeds is a common polysaccharide used in food, medicine, and regenerative treatments (Draget et al., 2002; D’Ayala et al., 2008; Sharma et al., 2023). Mannuronic and guluronic acid residues include carboxylic functional groups, making alginate pH-responsive (George and Abraham, 2006). Due to its anionic nature, alginate, its derivatives, and polyelectrolyte complexes are suitable carriers for pH-responsive drug delivery of many drugs such as famotidine, ampicillin, etc. (Jao et al., 2010; Modasiya et al., 2010). Alginic acid spontaneously forms gel in the presence of divalent cations such Ca^2+^, Ba^2+^, Sr^2+^, and Zn^2+^ (Shalaby and Burg, 2003). Alginate gels can encapsulate drugs or cells with minimal adverse effects due to their pH-dependent mild gelling ability (Milivojević et al., 2023).

Hyaluronic Acid (HA) is a anionic non cellulosic biomacromolecules (Duranti et al., 1998). Because the pKa of the carboxylic acid groups is between three to four, it behaves as an anionic polyelectrolyte at physiological pH, making it hydrophilic. It swells up to 1,000-fold compared to its volume due to its propensity to absorb water, resulting in a loose and water-rich network (Kamaly et al., 2016). Hydroxyl and carboxylic acid groups add functionality through conjugation, chemical bonding, and cross-linking, making pH-sensitive hydrogels easier to formulate (Kim et al., 2014; Kwon et al., 2015; Luan et al., 2017).

Dextran is a biocompatible and sustainable exo-polysaccharide produced by the bacterium *Leuconostoc mesenteroides*. The essential characteristic of dextran is prone to enzymatic degradation by dextranase. This enzyme is generated by a bacterium that belongs to the genus bacteroids found in the colon. Dextran is an exceptional carrier matrix for drug release in the colon as the enzyme dextranase breaks the 1,6-glycosidic bond of dextran and breaks down the hydrogel matrix (Hovgaard and Brøndsted, 1995; Simonsen et al., 1995; Chiu et al., 1999).

Xanthan, an anionic polymer with a carboxylic group on one of the side chains of glucuronic acid, is pH sensitive and exhibits swelling in the basic environment due to the carboxylic group ionization. The carboxylic acid group has a pKa value of 4.6, above which it is ionized with a negative charge, causing swelling. As a result, it can be employed as a pH-responsive hydrogel in the intestinal region at pH 7.4 for controlled drug delivery above its pKa value (Bueno et al., 2013).

Guar gum contains acidic functional groups, which help in the expansion of hydrogel at intestinal pH of 7.4. It is sensitive to microbial degradation in the large intestine, (Sinha and Kumria, 2001; Seeli and Prabaharan, 2016). Because of its chemical stability over a wide pH range, controlled drug release, and, most crucially, microbial breakdown in intestinal fluids, the hydrophilic characteristic of guar gum can be used for oral and colon-specific drug delivery (Seeli and Prabaharan, 2016). In one study, drug release was sustained in simulated gastric and intestinal fluids up to 20%, but in simulated colonic fluid increased up to 80%–100%. The presence of the enzyme galactomannanase increased tablet disintegration time, subsequently leading to more rapid, ultimately drug release from the formulation (Wong et al., 1997).

Guar gum succinate is a derivative of guar gum used for colon-focused drug delivery due to hydrophilicity, pH responsiveness, sustained drug delivery, and susceptibility to microbial degradation. Guar gum succinate microparticles were developed as a pH-responsive colon-specific medication carriers. The performance of these particles in physiological fluids was studied, and it was shown that at pH 7.4 in simulated intestinal fluid (SIF), the microparticle swelled faster and exhibited rapider drug release compared to simulated gastric fluid (SGF) pH 1.2 (Seeli et al., 2016).

Carrageenan is the name of the family of red seaweeds. It is the major component of red seaweed cell walls. Carrageenan is a polysaccharide composed of alternating units of -D-galactose and -D-galactose or 3,6-anhydro-D-galactose linked by −1,3 and −1,4-glycosidic links. Carrageenan is classified into six separate subtypes: kappa, lambda, nu, iota, theta, and mu. The biological form mu and nu carrageenan are precursors for kappa and iota carrageenan, respectively, whereas theta carrageenan is physiologically generated from lambda carrageenan (Campo et al., 2009). Generally speaking, kappa carrageenan is used as a pH responsive material. Water-soluble carrageenan’s are insoluble in organic solvents. The quantity of sulphate substitution and the equilibrium of related cations determines carrageenan aqueous solubility, viscosity, and gel strength (Li et al., 2014). Carrageenan’s anionic nature makes it suitable to use in pH-sensitive hydrogels for controlled medication release systems. In a previous study, the drug release rate increased from 15% at pH of 1.2%–80% with the in pH of 7.4 of the medium (Kotwal et al., 2007).

Chondroitin sulfate (CS) is water soluble natural polysaccharide polymer composed of D-glucuronic acid and N-acetyl-D-galactosamine. Due to the carboxylic and sulphonate functional groups, CS shows increased water uptake capacity (Suhail et al., 2022). Fucoidan, a long chain anionic polysaccharide with sulphate functional group, is present in some species of brown algae. The seaweed species *Fucus vesiculosus, Cladosiphon okamuranus, Laminaria japonica,* and *Undaria pinnatifida* are frequently used to extract the fucoidan used in commercial products. Due to its large number of negatively charged sulfonic acidic groups, fucoidan is considered to be pH sensitive (Wang et al., 2022; Haggag et al., 2023). Galactommans are polysaccharides-based macromolecules obtained from plants, microorganisms, animals and algae. It shows no intrinsic gelling abilities but is commonly used in synergy with other gelling agents such as xanthan gum, agar gum, etc. (Silveira and Bresolin, 2011).

## 3 pH-sensitive hydrogel in drug delivery applications

pH-sensitive hydrogels have been extensively explored and employed in drug delivery systems that take advantage of pH variations along the GIT.

Bio-macromolecules responsive to changes in the local physiological environment govern cellular processes in biological systems (Shakya et al., 2010). In the human body, pH ranges from extremely acidic, i.e., 1.2 to normal, i.e., 7.4 (Florence and Attwood, 1998). pH differences can also be found in within organs like the vaginal tract, gastrointestinal tract, blood vessels, etc., extracellular and endosomal/lysosomal microenvironments, and the skin (Lee et al., 2007; Bae et al., 2012; Koetting et al., 2015). Furthermore, the pH variations between normal and malignant tissues is used in the disease’s treatment solutions (Ko et al., 2007). In comparison to normal tissues, the tumor microenvironment is highly acidic due to tumor cells’ aggressive metabolism (Liu et al., 2014). pH-sensitive hydrogels are widely utilized in medicine or gene delivery to target specific organs or regions in the human body and cancer treatment to assault malignant cells (Zhuo et al., 2020).

Two basic approaches are used in the formation of pH-responsive drug delivery systems. Firstly, polymers with ionizable groups are used to fulfill the organ- or site-based approach. These ionizable groups can achieve a targeted and regulated drug discharge in reaction to local pH alterations by experiencing conformational, solubility changes or transitioning between swelling and deswelling states (Kocak et al., 2016; Deirram et al., 2019). The second method involves forming ionic interactions between the pH-responsive polymer and the drug. The ionic interaction can dissolve in response to pH changes, causing the drug to be released from the polymer backbone (Pang et al., 2016; Deirram et al., 2019). Nanoparticles, nanoaggregates, nanogels, nanocapsules, core-shell particles, micelles, liposomes, polymersomes, hydrogels, layer-by-layer films, and bioconjugates have been developed as pH-sensitive polymer-based drug delivery devices (Hendi et al., 2020; Li et al., 2021; Ofridam et al., 2021).

### 3.1 Oral drug delivery

Drugs that are absorbed poorly along the GIT and higher pH are perfect candidates for stomach delivery. Gastric specific delivery is critical for gastric cancer, gastritis, and carcinoma (Ishak et al., 2007). Some of the above-mentioned stomach disorders are caused by *Helicobacter pylori*, which inhibits the gastric mucus layer. Due to insufficient drug absorption beyond the gastric mucus barrier and poor chemical stability in the gastric medium, single antibiotic therapies may not appear effective. Chitosan, its derivatives, and blends are employed as successful carriers to increase the drug residence time in the stomach and its sustained release (Bami et al., 2022). In one study, pH-sensitive chitosan-based hydrogels loaded with metronidazole were prepared. The hydrogels swelled more rapidly at the stomach pH and showed increased drug release rate compared to the intestinal pH. These findings revealed prolonged residence time of hydrogel systems in a dog’s stomach of over 48 h compared to commercially available metronidazole tablets. The dog’s stomach contained pH-sensitive chitosan-based hydrogels for 48 h, as seen by radiography. This shows that the housekeeping wave did not interchange the hydrogel. The migrating mylo-electric cycle of the stomach emptying cycle, which happens every 2 h in humans and 1 h in dogs, did not empty the hydrogel. The hydrogel turned around in the stomach on the radiographs, indicating that it floated on the gastric fluids rather than adhering to the gastric mucosa (El-Mahrouk et al., 2016).

Orally administered drugs are mainly absorbed in the small intestine. The primary goal of oral drug delivery is safely delivering the drugs from the stomach’s acidic environment to the weakly alkaline intestinal milieu while preserving their pharmacological activity. Poor permeability through the intestinal mucosa, acid-catalyzed drug degradation, and proteolytic degradation along the GIT are additional challenges in oral drug delivery, along the pH gradient ranging from pH 1.2 (stomach) to pH 8 (intestine) (George and Abraham, 2007). These issues are addressed mainly by preventing drug release in the stomach by employing hydrogel carriers that shrink in the acidic gastric environment, preventing the drug from being released. Natural polymers with anionic pendant groups like carboxylic and sulphonate functional groups that remain protonated in an acidic medium the best choice for hydrogel delivery system matrix. As a result, hydrogels maintain their shrunk condition in this acidic environment, preventing rapid drug release. Acrylic acid and its derivatives are commonly used in naturally grafted polymers to counteract pH sensitivity and deliver the drug from the acidic gastric environment to the intestine, where the highest amount of drug can be released and absorbed (Yang et al., 2016).

Dexamethasone has a short half-life (2-5 h) in the plasma due to the low aqueous solubility. Due to short half-life and indicated to be used in ulcerative colitis, it must be administrated in the small intestine and colon. Acrylic acid grafted guar gum/β-cyclodextrin composite hydrogels linked with tetraethyl orthosilicate (TEOS) for targeted intestine administration of dexamethasone were successfully formulated for this purpose. The hydrogels were pH-sensitive, with delayed drug release correlating to guar gum concentration and the highest drug release achieved at high pH in the colon (Das and Subuddhi, 2015).

Therapeutic delivery into the systemic circulation via colonic absorption is a unique method of delivering peptide and protein therapeutics, as well as drugs that are poorly absorbed via the upper gastrointestinal GIT. Colonic drug delivery is beneficial for treating arthritis, angina, nocturnal asthma, and colonic disorders such as colorectal cancer, Crohn’s disease, and ulcerative colitis (Arévalo-Pérez et al., 2020; Lee et al., 2020; Naeem et al., 2020; Cui et al., 2021).

pH-responsive hydrogels prone to microbial breakdown have an exceptional property that allows controlled and drug delivery in the colon. Guar gum and its analog-based composite hydrogels are preferred materials for sustained drug delivery in the large intestine, which is accompanied by guar gum-based hydrogels natural microbial breakdown. pH-responsive hydrogels beads, made of guar gum succinate-sodium alginate (GGS-SA), were prepared to develop ibuprofen-controlled release formulations. The hydrogel beads swelled faster and released more ibuprofen at pH 7.4 than compared with pH 1.2, respectively. The reason behind this were the anionic groups on the side alginate side chains, while the sustained drug release was achieved due to guar gum succinate swelling properties. (Seeli et al., 2016).

Insulin oral delivery has numerous hurdles due to its chemical structure. Oral insulin administration has several benefits *such as* fast hepatic insulin delivery, avoidance of peripheral hyperinsulinemia, weight gain, hypoglycemia, and improved patient compliance (Fonte et al., 2013). However, intestinal proteses degrade insulin, and its permeability through the intestinal wall is poor due to relatively high molecular weight and high hydrophilicity. Various drug delivery techniques, such as pH-responsive hydrogels, microparticles, nanoparticles, permeation enhancers, and insulin conjugates, have been developed to address these issues (Ramesan and Sharma, 2014). Due to obvious shortcomings of invasive drug delivery like lipoatrophy, lipohypertrophy, poor patient compliance, sterile manufacturing, painful administration, high manufacturing costs, oral insulin delivery is preferred. Insulin is administrated into systemic circulation directly by injections, generating peripheral hyperinsulinemia, which leads to hypoglycemia, cancer, atherosclerosis, and peripheral hypertension (Lee et al., 2010).

Many researchers have investigated pH-responsive hydrogels for oral insulin delivery to bypass its degradation by intestinal proteses like trypsin, alpha-chimotrypsin and elastase breakdown in the stomach’s acidic medium, by leveraging the anionic hydrogels natural propensity of swelling exclusively in the alkaline environment of the intestine. Mukhopadhyay, Piyasi, et al. (2014) formulated a pH sensitive hydrogel of insulin for oral delivery using N-succinyl chitosan. Developed pH sensitive hydrogel released the insulin in stomach was negligible while the release in alkaline environment was quantitive. Insulin-loaded hydrogel demonstrated strong hypoglycemic effects in diabetic mice following oral administration, with a 4.43% insulin relative bioavailability. Furthermore, *in vivo* toxicity and histopathology studies revealed no adverse effects (liver, kidney) after oral administration of the hydrogels (Mukhopadhyay et al., 2014). Cikrikci, S., Mert, B., and Oztop, M. H. (2018) was developed oral pH sensitivity gel of insulin using the alginate, gum tragacanth and chitosan and evaluated for *in vitro* drug release studies. Results indicated that hydrogel retain the insulin in gastric buffer and release in intestinal condition (Mukhopadhyay et al., 2014).

An aqueous free radical polymerization approach was employed to produce a drug-loaded smart hydrogel composed of chitosan and fenugreek-g-poly (MAA), which exhibits pH responsiveness. The network that was created underwent evaluation in several aspects including the percentage of capecitabine loading, swelling response, morphology, structural and compositional properties, and drug release behavior. The hydrogel formulations shown a notable increase in swelling and *in vitro* drug release rate when subjected to a pH of 7.4 compared to a pH of 1.2, thereby indicating the pH-responsive nature of the hydrogels. The swelling percentage and CAP loading exhibited a range of 74.45%–83.54% and 50.13%–72.43%, respectively. The hydrogels exhibited a regulated release pattern of capecitabine, with a maximum release of 93% observed within a 30-h timeframe. The improved formulation underwent further screening to assess its potential for acute oral toxicity in laboratory experiments. There were no observed indications of oral, cutaneous, or ocular toxicities, hence providing confirmation of the network’s safety profile. In addition, the pharmacokinetic research revealed the sustained release properties of capecitabine from hydrogels, as evidenced by a notable elevation in the plasma half-life (t1/2) (13 h) and the area under the curve (AUC) (42.88 μg h/mL) of capecitabine. Based on the aforementioned findings, it is highly advised to utilize manufactured hydrogels as a biocompatible carrier for the purpose of delivering active drugs to the colorectal region (Rehman et al., 2022).

### 3.2 Parenteral drug delivery

Hydrogels for injectable drug delivery have recently gained attention because of their potential to provide both rapid and sustained drug release following a single administration. This will reduce drug concentration and side effects (Rizwan et al., 2017). Ischemia, tumors, and healing wounds are all acidic environments (Xin and Naficy, 2022).

The production of injectable polysaccharide hydrogels with biocompatibility and self-healing properties has been achieved through the chemical crosslinking of multialdehyde guar gum (MAGG) and N, O-carboxymethyl chitosan (N, O-CMCS) using pH-sensitive, biodegradable, and dynamic Schiff base connections. This marks the first-ever instance of such crosslinking. The hydrogels exhibited favorable characteristics for the purpose of administering drugs by injection due to their remarkable viscoelastic, thixotropic, and self-healing qualities. After being loaded with Dox for a duration of 5 days, the hydrogels exhibited a release mechanism that was responsive to changes in pH. Notably, a greater amount of Dox was released at the acidic pH characteristic of tumor environments compared to the neutral pH typically found in healthy conditions. The non-toxicity of these hydrogels was confirmed by the use of MTT and hemolytic tests. The hydrogel loaded with Dox exhibited a substantial decrease in MCF-7 cell viability, resulting in a mortality rate of 72% (Shim et al., 2021; Garshasbi et al., 2023).

Liang, Yongping et al., 2019, was developed the injectable pH responsible hydrogel using the pullulan and chitosan. The effects of pH levels of 5.5, 6.8, and 7.4 on *in vitro* drug release were studied at 37°C. The extracellular matrix in tumor tissues often has a lower pH value than normal tissues due to acidification caused by glycolysis of tumor cells. At 5.5, the swelling ratio is larger and the DOX release percentage is higher than at 7.4, and this is because the electrostatic repulsion between the protonated -NH2 group in chitosan and the weaker connection between -NH2 and -CHO created during the Schiff base reaction causes the -NH2 group to repel the -CHO group (Rizwan et al., 2017).

Doxorubicin (DOX) is one of the most effective chemotherapeutical components for treating various types of cancer (Anand et al., 2018; Menendez et al., 2022). Omidi, S., Pirhayati, M., & Kakanejadifard, A. (2020), formulated the pH-sensitive injectable of doxorubicin and curcumin using a chitosan with graphene and cellulose nanowhisker (CGW). The hydrogel formulation exhibited a pH-responsive release pattern in relation to the delivery of anticancer drugs. The *in vivo* experiment demonstrated rapid gelation of the hydrogel upon subcutaneous injections into the skin of rats. The antimicrobial investigations conducted have confirmed that CGW exhibits robust antibacterial activity specifically against Gram-positive bacteria. The findings of this study indicate that the CGW hydrogel exhibits potential as a viable option for localized drug delivery systems (Omidi et al., 2020).

For efficient gene distribution, multifunctional gene vehicles using a layer-by-layer method with the top one as a pH-responsive hydrogel was explored. Condensing deoxyribonucleic acid (DNA) with protamine to form DNA/protamine complexes provided the cationic core that served as a template. With the help of a layer-by-layer approach, the anionic DNA, cationic liposomes, and the O-carboxymethyl Chitosan (CMCS-CLDPD complexes) were applied to the first layer. The top layer of CMCS hydrogel aids gene transfection efficiency while also protecting the CMCS-CLDPD from serum contact. Animal and cell culture studies revealed that the CMCS layer came off in the tumor medium at pH 6.5, allowing the loaded DNA to be released faster in the acidic tumor media than in the neutral condition (Li et al., 2012).

### 3.3 Transdermal drug delivery

The top layer of skin, namely, stratum corneum’s properties, such as cohesion, intercellular lipid, homeostasis, and permeation barrier are regulated by various circumstances, involving the skin’s pH. The pH of skin is between 5.0 and 6.0, so the stratum corneum is known as the acid mantle. Many factors influence the pH of the same, including age, gender, sebaceous glands, apocrine glands, eccrine glands, and epidermal cells. Skin sickness (acne, inflammation, and irritation), impaired permeation barrier, and cell cohesiveness in the acid mantle are symptoms of an imbalanced pH when the pH of the top layer of the skin is higher. Many biological polymers and their analogs, such as chitosan and carboxymethyl guar gum, have been utilized to encapsulate medicines because they produce free-standing membranes. To evaluate the medication transport of diclofenac, researchers developed nanosilica/acrylic acid grafted guar gum membranes for transdermal patch therapy. (GG-g-AA) stands for guar gum-g-acrylic acid. The composition 10/0.1/0.5 had the highest water uptake of all the compositions. Diclofenac release was more regulated in GG-g-AA nanocomposites than in guar gum (Hendi et al., 2020; Zhuo et al., 2020).

## 4 Biological fate of biopolymer hydrogel used in drug delivery

Biopolymer hydrogels employed in the context of drug delivery exhibit a distinct biological trajectory subsequent to their administration. Upon introduction into the biological system, the hydrogels undergo a process of swelling, resulting in the formation of a three-dimensional network architecture. Over a period of time, the materials undergo regulated biodegradation via enzymatic or hydrolytic mechanisms. The process of degradation results in the liberation of the encapsulated drug molecules into the immediate vicinity. The drugs that have been released adhere to their respective pharmacokinetic pathways, while the biodegraded components of the hydrogel are eliminated through diverse routes (Xin and Naficy, 2022). Mucoadhesion of non-cellulosic biological macromolecules is dependent on numerous mechanisms. These large molecules form mucoadhesive contacts with glycoproteins in the mucus layer via hydrogen bonding. The negatively charged mucin glycoproteins interact with the positively charged macromolecules via electrostatic forces. The hydrophobic contacts and chain entanglement caused by Van der Waals forces also play a role. Some macromolecules are able to penetrate the mucus layer and interact with underlying cells, while others can expand and create a gel to make close contact with mucosal surfaces. Collectively, these methods improve the mucoadhesion of non-cellulosic biological macromolecules, paving the way for their use in mucosal tissue-specific medication delivery (Zhuo et al., 2020).

## 5 Biopolymer based pH-sensitive hydrogel for drug delivery: opportunities and gaps

Biocompatibility, biodegradability, and non-toxicity are critical for biomedical and drug delivery materials. The pH, temperature, light, electrical or magnetic fields, and ionic strength of biological macromolecule-based materials have been studied. pH and temperature are two stimuli that exist naturally in the human body’s interior environment. Thus, internal stimuli-responsive hydrogels can deliver targeted medications via multiple channels. Non-cellulosic biological polymer-based pH-sensitive hydrogels have been widely researched for site-specific medication administration, cancer therapies, insulin delivery, and genetic material delivery. Smaller internal stimuli-responsive hydrogels are preferred for this reason. The pH-sensitive swelling/deswelling biological components were extracted or grafted or copolymerized with anionic/acidic monomers or cationic groups like quaternary ammonium groups on polymer chains (Zhuo et al., 2020). This strategies can help to develop commercially viable non cellulosic drug delivery systems. Different commercially available non cellulose based hydrogel products are shown in Table 2.

**TABLE 2:** Some commercially available non-cellulose based hydrogels. Adapted from (Cascone and Lamberti, 2020).

Main component	Commercial product	Company	Administration route
Hyaluronic acid	Gengigel^®^	Oraldent Ltd	buccal
	Vagisil^®^	Combe Inc	vaginal
	Zestica Moisture^®^	Searchlight Pharma Inc	vaginal
	Hyalo gyn^®^	Fidia pharma United States Inc	vaginal
Collagen and Sodium Hyaluronate	Collagen Hydrogel Mask	Skin Republic	transdermal
Sodium hyaluronate	Hylo^®^ Gel	Candorpharm Inc	ocular
non-animal stabilized hyaluronic acids	Juvederm^®^	Allergan	Parenteral (filler injections)
	Restylane^®^	Q-Med	Parenteral (filler injections)
bacterial-based hyaluronic acids	Captique^®^	Genzyme	Parenteral (filler injections)
Alginic acid	Ocusert^®^	Alza Corporation	Ocular
Alginate	Algisite M^®^	Smith and Nephew	Topical (Wound dressing)
	Medihoney^®^ Adhesive Dressings	Derma Sciences Inc	Topical (Wound dressing)
	Ca-alginate dressing	Gentell Corp	Topical (Wound dressing)
	NU-Derm^®^ Hydrogel	Systagenix	Topical (Wound dressing)
	Kaltostat^®^	Convatec	Topical (Wound dressing)
Xanthan gum	Buccastem^®^ M	Alliance	Buccal
Xanthan gum and gelatin	Nicotinell^®^	GlaxoSmithKline	Buccal

Chitosan, alginate, hyaluronic acid, guar-gum, and dextran are used most often in pH-sensitive hydrogels. The natural origin, weak mechanical properties, poor processability (cellulose and chitosan), limited sources, high production costs, rapid biodegradation, catabolization rates (e.g., during gelation), and susceptibility to microbial spoilage are all drawbacks of this material (Hendi et al., 2020). By studying effective extraction, tissue culture, biotechnology, and green chemistry, biological macromolecule synthesis and modification may be addressed. Efficient biological macromolecule extraction may lower manufacturing costs and microbial overburden. Development of high-yielding algae/plant strains, innovative biological macromolecules, and novel strains may overcome problems including poor processability, high biodegradation, supply constraint, and production costs. To solve these issues, future semi-synthetic and bioengineering solutions should physically, chemically, or biologically change biological macromolecule-based hydrogels. In addition, grafting techniques and copolymerization with other natural or synthetic molecules can open novel facets in the fields of pH responsive drug delivery (Bera et al., 2015; Chandel et al., 2016; Singh Chandel and Jewrajka, 2020). Recent pH-responsive hydrogel research, which uses non-cellulosic biological macromolecules, focuses on several drug delivery issues. First, new bio-derived macromolecules beyond cellulose may improve hydrogel performance, biocompatibility, and drug delivery. In addition, the incorporation of these hydrogels into state-of-the-art imaging and monitoring technologies, such as pH monitoring in real-time or drug administration guided by imaging, has the potential to enhance the accuracy and efficacy of therapies, therefore addressing significant deficiencies in existing medical methodologies (Ofridam et al., 2021).

The use of non-cellulosic macromolecules in biomaterials is consistent with global sustainability efforts. These materials are often renewable and may be made to biodegrade sustainably. Beyond scientific discoveries, this rise affects various sectors and economies. The improvement in biomaterial design allows researchers, physicians, and corporations to collaborate on novel medical devices and treatments. Large-scale biomaterial production might boost economic growth while addressing healthcare and environmental challenges. The development and design of biomaterials using non-cellulosic biological macromolecules present a significant chance to revolutionize the fields of medicine, technology, and sustainability. Overall non-cellulosic macromolecules based pH-stimuli responsive materials are a promising choice for targeted and sustained release drug delivery.
